# Projecting burden of hypertension and its management in Turkey, 2015-2030

**DOI:** 10.1371/journal.pone.0221556

**Published:** 2019-09-11

**Authors:** Ayda Aysun Yurekli, Nazmi Bilir, Muhammad Jami Husain

**Affiliations:** 1 Visiting Senior Research Scientist at UIC Illinois, Chicago, Illinois, United States of America; 2 Institute of Public Health, Hacettepe University, Ankara, Turkey; 3 Global Noncommunicable Diseases Branch, Division of Global Health Protection, Centers for Disease Control and Prevention, Atlanta, Georgia, United States of America; Heidelberg University, GERMANY

## Abstract

**Background:**

In Turkey, hypertension was responsible for 13% of total deaths in 2015. We apply existing research finding regarding the impact of a population-wide reduction in sodium consumption on the decrease of the hypertension prevalence rate among 15+ years population and the gender-age specific reduction in total death rates among 30+ years population, and compare hypertension burden, averted deaths, costs and benefits between two scenarios.

**Methods:**

The first scenario (i.e. status quo) assumes constant hypertension prevalence rate and the death rates between 2015 and 2030. Based on the Framingham Heart Study and INTERSALT Study findings on the impact of salt-reduction strategies on hypertension prevalence rate, the second scenario (Scenario II) assumes a 17% reduction in the prevalence of hypertension in Turkey in 2030, from its 2015 prevalence level. We project hypertension attributable disability adjusted life years (DALYs) in 2030, monetize DALYs using GDP (and income) per capita, and compare the projected economic benefits of DALYs averted and the additional costs associated with the increases in hypertension treatment through antihypertensive medications and physician consultations.

**Results:**

The estimated benefits of reducing the economic burden of hypertension deaths outweigh the cost of providing hypertension treatment. A decrease in hypertension prevalence by 17%, attributable to population-wide reduction in salt consumption, is projected to avert 24.3 thousand deaths in 2030. We projected that, compared to status quo, 392 thousand DALYs will be averted in Scenario II in 2030. The economic benefits of reduction in potential hypertension deaths are estimated to be 6.7 to 8.6 folds higher than the additional cost of hypertension treatment.

**Conclusion:**

Population-wide hypertension prevention and management is a win-win situation for public health and the Turkish health care system as the economic benefits of reducing deaths and disabilities associated with hypertension outweigh the costs significantly.

## Introduction

Hypertension, also known as high or raised blood pressure, accounts for about 10 million deaths worldwide each year [1]. Hypertension is a leading risk factor for cardiovascular diseases (CVD); 45% of deaths caused by ischemic heart disease and 51% of deaths caused by cerebrovascular disease are attributable to hypertension [1]. In Turkey, hypertension (high systolic blood pressure (SBP) ≥140 mm Hg) was responsible for over 54 thousand deaths in 2015, constituting 13.4% of total deaths. Among the Turkish population with hypertension in 2015, an estimated 25,663 deaths occurred from ischemic heart disease and 12,971 deaths from cerebrovascular diseases [2].

Global deaths from noncommunicable diseases (NCDs) will continue to rise over the next several decades due to aging populations [3, 4]. Evidence shows that hypertension also increases with age. Furthermore, behavioral (e.g., unhealthy diet, tobacco use, physical inactivity, harmful use of alcohol) and metabolic (e.g., obesity, diabetes, rising blood lipids) risk factors contribute to the development of hypertension and its health consequences [1, 5]. The changing demographic structures coupled with changing lifestyles (risky health behaviors) are significant health and economic challenge for preventing and controlling hypertension and its associated health risks worldwide and in Turkey.

Salt consumption, one of the leading causes of hypertension, is three times higher in Turkey than that of suggested per capita per day salt consumption level [6, 7]. The Turkish Government undertook a comprehensive salt reduction program in 2011 that includes the major components recommended by WHO for reduction of salt intake: monitoring, major reformulation actions, awareness, and interventions at the institutional settings [6, 8]. Existing studies find that a population-wide modest reduction in sodium consumption can reduces blood pressure to a level that leads to the reductions in hypertension prevalence rate, and in total deaths. For example, Cutler et al. [9] suggested that an average reduction of 77 mmol per day in dietary intake of sodium resulted in a 1.9 mmHg systolic and 1.1 mmHg decline in diastolic blood pressure. Research based on INTERSALT STUDY also reveals that if a population, on average, had a daily sodium intake lowered by 100 mmol (about one teaspoon salt), average population systolic blood pressure (SBP) would be lower by 2–3 mm Hg. For each 2-mmHg difference in SBP, death from coronary heart disease will decline by 4%, stroke by 6% and all deaths by 3%. Furthermore, a 5 mmHg lower SBP will reduce the death by 9% for coronary heart disease, 14% for stroke and 7% for all deaths [10]. Stamler [10] argued that mortality reduction of 3% or 5% magnitude can be achieved if the sole approach were to intensively treat the hypertension patients in that population with antihypertensive medication. In an analysis based on Framingham Heart Study, Cook et al. [11] revealed that a 2-mmHg reduction in blood pressure will reduce the hypertension prevalence by 17% for 35–64 years of age US residents [11, 12]. When the salt reduction is combined with a healthy diet (DASH diet), blood pressure is lowered by 11.5 mmHg among people with hypertension [13].

In this paper, we apply existing research finding regarding the impact a population-wide reduction in sodium consumption on the decrease of the hypertension prevalence rate among 15+ years population and the gender-age specific reduction in total death rates among 30+ years population, and compare hypertension burden, averted deaths, costs and benefits between two scenarios. The first scenario (i.e. status quo) assumes constant hypertension prevalence rate and the death rates between 2015 and 2030. The second scenario (scenario II) assumes a 17% reduction in the prevalence of hypertension in Turkey in 2030, from its 2015 prevalence level. In the spirit of the Framingham Heart Study finding by Cook et al. [11], the reduction in death rates is assumed to be attributable to the salt reduction program by Turkey Ministry of Health (MoH) and associated population level reduction in the level (mmHg) of blood pressure by age and gender. We project hypertension attributable DALYs in 2030, monetize DALYs using GDP (and income) per capita, and compare the projected economic benefits of DALYs averted and the additional costs associated with the increases in hypertension treatment through antihypertensive medications and physician consultations.

## Background

As Turkey’s population has been increasing, it is also getting older. It is projected that population will increase by 13%, from 78 million in 2015 to 88 million in 2030, where 12.1% of the population will be age ≥65 years in 2030, up from 7.8% in 2015 [14]. As the population ages, World Bank projects that female life expectancy will increase from 78.7 years in 2015 to 81.9 years in 2030; and the male life expectancy will increase from 72.2 years in 2015 to 76 years in 2030 [14]. Risky behavior and metabolic risk factors that influence hypertension are also high in Turkey. In 2015, an estimated 27.9% of the Turkish population aged ≥20 years was obese (*BMI* ≥ 30)—more in women (34.7%) than in men (20.6%) [2]. The level of salt consumption among the Turks is one of the highest in the globe; on average 15 grams salt or 5,895 mg of sodium per day in 2012; higher in men (15.7 grams salt or 6,178 mg sodium per day) than in women (14.0 grams salt or 5,510 mg sodium per day) [6, 15]. Turkey is still among the top countries with high smoking prevalence rates (i.e., 27.1% among adults aged ≥15 years in 2012) [16, 17]. Dastan et al. [18] examined the risk factors that influence hypertension in Turkey by analyzing Chronic Risk Factors Survey 2011 and found socioeconomic and modifiable risk factors associated with hypertension in both genders, including lack of awareness and not being in treatment, lower education and sedentary lifestyle in women, and unhealthy dietary habits, smoking and alcohol consumption in men. Furthermore, the study found that having diabetes and being obese are positively correlated with the poor control of hypertension [18].

Some nationwide surveys between 1998 and 2012 examined the hypertension prevalence and management in Turkey, including the Chronic Diseases and Risk Factors Survey (CDRFS) [19]; TURDEP I and TURDEP II [20, 21]; and PatenT I and PatenT II [22, 23, 24] (Table 1). According to the Chronic Diseases Risk Factors Survey (CDRFS) conducted by the Ministry of Health in 2011, about 24% (or 13.5 million) adult population aged ≥15 years had hypertension. Prevalence of hypertension was more in women (26% in women versus 24% in men) and increased with age, such that about 7 in 10 adults >65 years of age have hypertension [19].

**Table 1:** Surveys of hypertension prevalence in Turkey.

	TURDEP I^1^	PatenT I^2^	TURDEP II^1^	CDRFS^3^	PatenT II^2^
Year	1998	2003	2010	2011	2012
Age group	≥20	≥18	≥20	≥15	≥18
Prevalence (crude) %	29	31.8	31.3	24	30.5
Prevalence (crude) % by gender	M: 25.6	M: 27.5	M: 30.9	M: 24	M: 28.4
	W: 31.3	W: 36.1	W: 32.3	W: 26	W: 32.3

Source:

^1^ Satman et al., 2002 [20] and Satman et al. 2013 [21];

^2^ Altun et al., 2005 [22], PatenT I [23] and Paten T II. 2012 [24],

^3^ Republic of Turkey Ministry of Health (2013) Chronic Diseases and Risk Factors Survey in Turkey [19].

M: Men; W: Women. Surveys identify hypertensive people: (i) if they have not been diagnosed with hypertension, but their average blood pressure (BP) measurement is equal or higher than ≥140 mmHg for systolic blood pressure (SBP) or ≥90 mmHg for diastolic blood pressure; (ii) if they have been diagnosed with hypertension before and have used antihypertensive medication, regardless of their blood pressure measurement; and (iii) if they have been diagnosed with hypertension but have not used any medication, but their average BP measurement indicates SBP/DBP ≥140/90 mmHg.

Estimates reveal that the number of deaths and the death rates associated with hypertension has declined over the period 1990 to 2005; a 5.1% decline in the number of deaths from 56,392 in 1990 to 53,493 in 2005; and 36% fall in death rates– 234.2 deaths to 140.3 deaths per 100,000 population age 25+ years old; respectively [2]. In recent years, hypertension control initiatives such as improving awareness, treatment, and control of high blood pressure increased in Turkey. For instance, 2003 PatenT I and 2012 PatenT II surveys reveal that hypertension awareness (i.e., the percentage of hypertension cases diagnosed by a physician) increased from 40% in 2003 to 54.7% in 2012. Between 2003 and 2012, awareness was higher, and increased more, in women (48% and 70%) than in men (28% and 41%), respectively. Furthermore, nationwide surveys report increases in antihypertensive treatment among people with hypertension (Table 2) [22, 23, 24, 25]. For example, in 2012, 47.5% people with hypertension received antihypertensive treatment–up from 31% in 2003.

**Table 2:** Trends in awareness, treatment, and control of hypertension*.

	TURDEP I^1^ 1998 ≥20 age	PatenT I^2^ 2003 ≥18 age	HINT^3^ 2007 ≥18 age	TURDEP II^1^ 2010 ≥20 age	CDRFS^4^ 2011 ≥15 age	PatenT II^2^ 2012 ≥18 age
Awareness**	44.6%	40%		58.1%	63%	54.7%
Treatment***	45.3%	31%	40%		57.5%	47.5%
Control****	30%	8%	13.6%		30%	28.7%

Source:

^1^ Satman et al., 2002 [20] and Satman et al. 2013 [21];

^2^ Altun et al., 2005 [22] and PatenT I [23] and Paten T II 2012 [24],

^3^ Arici et al., 2010 [25]

^4^ Republic of Turkey Ministry of Health (2013) Chronic Diseases and Risk Factors Survey in Turkey [19].

* Adults at given age are diagnosed with high blood pressure with SBP ≥140 mmHg for systolic blood or ≥90 mmHg for diastolic blood pressure (DBP) by a physician or by the measurement taken during the survey. Also, people who currently use antihypertensive medication are considered hypertensive regardless of their blood measures taken during the survey.

**Awareness includes adults who have been diagnosed previously by a physician as hypertensive and during the survey their measurement shows SBP and DB>140/90 mmHg.

*** Treatment includes adults who have diagnosed previously as hypertensive by a physician and have been using antihypertensive medication.

****Control includes adults who have been diagnosed previously as hypertensive by a physician, but currently, they have not been using antihypertension medication because their blood pressure is SBP/DP<140/90 mmHg.

Turkish MoH survey (CDRFS 2011) revealed higher awareness (63%) among people with hypertension and relatively higher percentage (57.5%) under the hypertension treatment. However, only 30% of the people who are under treatment maintains hypertension under control. Furthermore, among those under treatment, 63% also have prescription medication for chronic diseases [19]. However, the improvements in awareness and the reduction in the number and the rates of death associated with hypertension in Turkey are leveled off in recent years. The estimated death rate due to hypertension has increased by 14% from 60.8 deaths per 100,000 population in 2010 to 69.4 deaths in 2015; and the number of hypertension deaths has also increased by 23% from 44 thousand to 54 thousand, respectively. Consequently, hypertension claims a higher percentage (13.4%) of total deaths in 2015 compared to that (12%) in 2010 [2, 26]. There has been global calls for investing in hypertension prevention and control initiatives. Evidence suggests that the benefit of reducing the burden of hypertension outweighs the costs of providing blood-pressure-lowering drugs to hypertensive adults. For example, providing antihypertension medication to 60% of the hypertensive population in Bangladesh generates 12.7 to 1 annual return on investment by 2021 and 8.6 to 1 annual return on investment by 2030 [27]. In a global and regional study on the effectiveness and costs of interventions to lower systolic blood pressure and cholesterol, Murray et al. (2003) asserted that non-personal health interventions, including reduction in the salt content of processed foods, are cost-effective ways to limit cardiovascular disease and could avert over 21 million DALYs per year worldwide [28].

### Data

Data on hypertension prevalence, treatment, control, and the awareness by age groups and gender are obtained from the published report by the Ministry of Health (MoH) based on the 2011 Chronic Diseases Risk Factor (CDRFS) survey, which is the first nationwide cross-sectional survey of MoH to monitor chronic diseases and their risk factors. The Institute for Health Metrics and Evaluation (IHME) Global Burden of Death (GBD) database provided Turkish data for the 2015 life expectancy, number of deaths, the years of life lost (YLL) due to premature mortality, the years lost due to disability (YLDs) and the disability adjusted life years (*DALY*) associated with hypertension (SBP140≥ mm Hg) by age groups and gender. We obtained age group and gender specific total annual population for 2015 and 2030 from the World Bank Health, Nutrition and Population (HNP) database, and the total number of deaths in 2015 for age groups by gender by the Turkish Statistical Institute (TUIK). Data on antihypertension medication usage for 18+ age population was obtained from a published nationwide survey PATENT II (2012) report [24]. We collected the prices of most common antihypertension medications from randomly selected pharmacies in Ankara Turkey in 2016 and confirmed those prices with the Turkish Pharmaceutical Association. National health insurance was implemented in 2011 in Turkey. Both private and government insurance plans cover as much as 90% of the population where about 90% have government health insurance. Since the government reimburses a significant portion of prescription drugs’ costs to pharmacies, the drug prices are controlled and do not vary by location across Turkey. We obtained the data for 2015 health care expenditures for ambulatory care services and the gross income per capita by gender from the Turkish Statistical Institute (TUIK). Finally, we used the International Monetray Fund (IMF) World Economic Outlook database for the gross domestic product (GPD) per capita, GDP deflator, and the consumer price index (CPI), including the projected values beyond 2017.

### Methodology

#### Projecting total deaths and hypertension attributable deaths in 2030

We projected total deaths from all causes in 2030 using the 2015 data on population and total deaths by gender and age-groups. First, we derived the weighted total death rates for male and female population in 2015.

$${{WDr}_{AC}\;}_{15}^{k}\;=\;\sum {{Dr}_{AC}\;}_{15}^{ik}*{shPop\;}_{15}^{ik}$$ (1)

${{Dr}_{AC}}_{15}^{ik}$ is the share of all cause deaths in population by gender (*k)* and five years age-groups starting 30–34 years to 80+ years of age (*i*) in 2015 and ${shPop\;}_{15}^{ik}$ represents the 2015 population shares by age-groups and gender. The use of the weighted total death rate (${{WDr}_{AC}\;}_{15}^{k}$) ensures the distribution of age-gender specific number of deaths based on their shares in total population. Assuming that ${{WDr}_{AC}\;}_{15}^{k}$ will remain constant between 2015 and 2030, we projected the total deaths for male and female population (30+ age) in 2030 using Eq 2.

$$\hat{{({Death}_{AC}\;}_{30}^{k})}\;=\;{POP\;}_{30}^{k}*\;{{WDr}_{AC}\;}_{15}^{k}$$ (2)

$\hat{{({Death}_{AC}\;}_{30}^{k})}$ is the projected total deaths from all causes in 2030 for male and female population aged 30+. We assumed that the age-group specific shares of total deaths among 30+ years of age population in 2015 (${{\%D\;Share}_{AC}\;}_{15}^{ik})$ will remain constant under the status quo condition, and distributed the gender-specific projected total deaths in 2030 among the age-groups in Eq 3.

$${\hat{{Death}_{AC}\;}}_{30}^{ik}=\hat{{({Death}_{AC}\;}_{30}^{k})}*{{\%D\;Share}_{AC}\;}_{15}^{ik}$$ (3)

We projected the gender and age-group specific hypertension (HBP) deaths in 2030 using similar method and assumptions described above, and as shown below in Eqs 4–6.

$${{WDr}_{HBP}\;}_{15}^{k}\;=\;\sum {{Dr}_{HBP\;}}_{15}^{ik}*{shPop}_{15}^{ik}$$ (4)

$$\hat{{{Death}_{HBP}\;}_{30}^{k}}\;=\;{({WDr}_{HBP\;}}_{15}^{k})*{POP\;}_{30}^{k}$$ (5)

$${\hat{{Death}_{HBP}}\;}_{30}^{ik}\;=\;\hat{{({Death}_{HBP\;}\;}_{30}^{k})}*{{\%D\;Share}_{HBP}\;}_{15}^{ik}$$ (6)

Where, ${{WDr}_{HBP}\;}_{15}^{k}$ is weighted hypertension attributable death rates for male and female population in 2015;${\;{Dr}_{HBP}}_{15}^{ik}$ is the share of hypertension deaths in population by gender (*k)* and age groups (*i*) in 2015, $\hat{{({Death}_{HBP}\;}_{30}^{k})}$ is the projected total deaths from hypertension in 2030 for male and female population aged 30+, ${\hat{{Death}_{HBP}}\;}_{30}^{ik}$ is the gender and age-group specific hypertension (HBP) deaths in 2030, and (${{\%D\;Share}_{AC}\;}_{15}^{ik}$) is the age-group (30+ years) specific shares of hypertension deaths (30+ years) in 2015.

We derived a second set of projections (i.e. Scenario II) for the total deaths and hypertension deaths in 2030. We assumed that Turkey will reduce population-wide salt consumption by 2030 with a comprehensive salt reduction program, contributing to the reductions in death rates and prevalence rates. The INTERSALT study (Stamler, 1991) revealed that if a population-wide reduction of sodium intake reduces on average 100 mmol (about 1 teaspoon) daily, average population systolic blood pressure (SBP) would be lower by 2–3 mmHg over 6 to 19 years [10]. A 2 mmHg, 3 mmHg, and 5 mmHg reductions in SBP could lead to 3%, 5%, and 7% reductions in total deaths, respectively [10]. Asaria et al. (2007) investigated how many deaths could potentially be averted over 10 years (2006–2015) by implementing a population-wide salt reduction by 15% in 23 countries, including Turkey; and provided estimates for the reduction in salt intake (gram per day) by 5 year age groups starting 30 years of age and the corresponding decrease in mean SBP (mm Hg) in 2015 [29]. Table 3 shows the finding from these two studies.

**Table 3:** Population-wide reduction in salt intake and the associated reduction in mean SBP by age groups in 2015, Turkey.

**Potential for lowering mortality with lower average population SBP mmHg** **(Source: Stamler, 1991)** [10]						
Reduction in SBP	2 mmHg		3 mmHg		5 mmHg	
Reduction in all deaths	-3%		-5%		-7%	
**Population-wide reduction in salt intake and the associated reduction in mean SBP by age groups** **(Source: Asaria et al., 2007)** [29]						
**Age groups**		**30–44**	**45–59**	**60–69**	**70–79**	**80+**
Reduction in salt intake (gram per day)	Male	2.2	2.4	2.4	2.4	2.4
	Female	1.5	1.7	1.7	1.7	1.7
The associated reduction in mean systolic blood pressure by 2015 (mm Hg)	Male	1.4	2.0	2.9	3.6	4.5
	Female	1.0	1.8	2.5	3.0	3.6
**Projections for 2030 (Scenario II) deaths from all-causes** (% *Reduction in Death*_*AC*_^*ik*^)						
**Age groups**		**30–44**	**45–59**	**60–69**	**70–79**	**80+**
Percent reduction in total death from all-causes	Male	2.1%	3.0%	4.6%	5.5%	6.5%
	Female	1.5%	2.7%	4.0%	5.0%	5.5%

In our 2030 projections under Scenario II, we combined the findings from Stamler (1991) and Asaria et al. (2007) and calculated a set of values for gender and age-group specific percent reductions in total deaths from all causes. For this, based on Asaria et al. (2007) we assigned 6% reduction in deaths for the 4 mmHg reduction in SBP (i.e. the average of the reductions in deaths between 3mm Hg and 5 mm Hg reduction); calculated the percent reduction in death per mmHg reduction of salt intake at 2, 3, 4, and 5 mmHg; assigned interpolated values for in-between mmHg reductions by taking averages of the reduction in death (%) per mmHg reduction in salt; and then derived the gender and age-group specific percent reductions in total deaths. The bottom panel in Table 3 shows the percent reduction in total death from all-causes used in this study for the 2030 Scenario II projections, which are based on the findings from Stamler (1991) and Asaria et al. (2007) in the top and middle panel.

Accordingly, the projected total deaths from all causes in 2030 in Scenario II is derived from the corresponding total deaths that were predicted under status quo condition in 2030, as displayed in Eq 7 below.

$$\overset{̿}{{{Scenario\;II\;Death}_{AC\;}}_{30}^{ik}}\;=\;{\hat{{({Death}_{AC}\;}_{30}^{ik})}*(1-({\%\;reduction\;in\;Death}_{AC}}^{ik}))$$ (7)

We assumed that population-wide averted deaths due to salt reduction will occur among the people with hypertension and estimated the Scenario II hypertension deaths in 2030 using Eq 8.

$$\overset{̿}{{{Scenario\;II\;Death}_{\;HBP\;}}_{30}^{ik}}\;=\;\hat{{{Deaths}_{HBP}}_{30}^{ik}\;}-[(\hat{{{Deaths}_{AC\;}}_{30}^{ik}\;)}-\hat{{({Scenario\;II\;Deaths\;}_{AC\;}}_{30}^{ik})]}$$ (8)

#### Projecting the disease burden associated with hypertension in 2030

Since the inception of the global burden of disease (GBD) study in 1993, disability-adjusted life years (DALYs) have been used extensively to provide a comprehensive assessment of the burden of disease and injuries, at global, regional and country level [30, 31, 32]. We projected the burden of hypertension in terms of DALYs, which is comprised of the years of life lost (YLL) due to premature mortality and the years lost due to disability (YLD).

$${DALY}_{t}^{ik}\;=\;{YLL}_{t}^{ik}+{YLD}_{t}^{ik}$$ (9)

Where (*i*) refers to the 5-years age-group starting 30–34 years until 80+; *k* is the gender (male and female), and *t* represents the years 2015 and 2030.

We projected the gender-specific *YLL*_30_ as the following.

$${\hat{\;YLL}\;}_{30}^{ik}\;=\;{E(x)}_{15}^{ik}*\;{\hat{{Death}_{HBP}}\;}_{30}^{ik}\;(Status\;quo\;scenario)$$ (10)

$$\overset{̿}{{YLL}_{30}^{ik}}\;=\;{E(x)}_{15}^{ik}*\overset{̿}{{{Scenario\;II\;Death}_{\;HBP\;}}_{30}^{ik}}\;(Scenario\;II)$$ (11)

The 2015 life expectancies ${E(x)}_{15}^{ik}$ at age-groups (*i*) were kept constant until 2030 for two reasons. First, 2015 YLLs, YDLs, and the DALYs were estimated by using 2015 life expectancy by IHME. Second, the life expectancy in 2015 was slightly higher than the life expectancy estimates by other organizations, notably by TUIK and the World Bank for the same year. IHME estimates for 2015 was also comparable with the World Bank’s projected life expectancy for Turkey in 2030. We applied the technique used by Norman et al. [33] and the GBD 2004-updates (WHO 2008, Appendix B) [34] to project the years lost due to disability ${\hat{YLD}}_{30}$ for 2030 status quo as the following.

$$\hat{{YLD\;}_{30}^{ik}}\;=\;{\hat{YLL}\;}_{30}^{ik}*(\frac{{YLD\;}_{15}^{ik}}{{YLL\;}_{15}^{ik}})$$ (12)

Where we obtained ${\hat{YLL}\;}_{30}^{ik}$ from Eq (10) and estimated $\left(\frac{{YLD}_{15}}{{YLL}_{15}}\right)$ from the IHME data in 2015. We obtained the projected population for age groups by gender in 2030 (*Pop*_30_) by the WB HNP database. We replaced Eqs (10) and (12) into Eq (9) and projected $\hat{{DALY}_{30}^{ik}}$ for the status quo. For the Scenario II, the projected YLDs were estimated by the following.

$$\overset{̿}{{YLD\;}_{30}^{ik}}\;=\;\overset{̿}{{YLL}_{30}^{ik}}*(\frac{\hat{{YLD\;}_{30\;}^{ik}}}{\hat{{YLL\;}_{30}^{ik}}})$$ (13)

#### Projecting the number of people with hypertension in 2030

Cook et al. (1995) study showed that a modest 2-mm Hg reduction in diastolic blood pressure (DBP) among population 35–64 years would reduce the prevalence of observed diastolic hypertension by 17%; and asserted that a combination strategy of a population-wide reduction in DBP and targeted medical intervention is most effective and could double or triple the impact of medical treatment alone [11]. We assumed same prevalence of hypertension for the years 2015 and 2030 under status quo. In Scenario II, we projected the number of people with hypertension in 2030 by gender and age-groups, assuming a 17% reduction in the prevalence rate in 2030 compared to 2015 level. In Turkey, since the inception of the universal health coverage in 2010, medical interventions (physician/hospital visits and prescription drugs) have been heavily subsidized by the government. Considering one of the objectives of the study is to estimate the benefits of reduced hypertension due to salt reduction and the medical interventions (increases in antihypertensive medication usage), a 17% reduction in hypertension prevalence between 2015 and 2030 would be conservative. Furthermore, the benefits of a 17% reduction in hypertension prevalence due to salt reduction and medical interventions by 2030 would be consistent with the assumptions used for the cost projections of hypertension deaths by 2030.

In the status quo, we projected hypertension cases and the rates by multiplying gender and age-group specific prevalence rates in 2030 by corresponding age-gender populations in 2015 and 2030.

$${HBP\;cases\;}_{15}^{ik}\;=\;{Prev\;Rate}_{15}^{ik}*{Pop\;}_{15}^{ik}$$ (14)

$$\hat{{HBP\;cases\;}_{30}^{ik}}\;=\;{Prev\;Rate}_{15}^{ik}*{Pop\;}_{30}^{ik}$$ (15)

For the scenario II, where we assume 17% reduction in the prevalence rate in 2030, we first estimated weighted prevalence rate by gender in status quo by the following:

$$\hat{{{WPrev\;}_{HBP}\;}_{30}^{k}}\;=\;\sum \hat{{{Prev\;}_{HBP\;}}_{30}^{k}}*{{\;\%Share}_{POP\;}}_{15}^{ik}$$ (16)

Where,

$${\hat{\;{Prev}_{HBP}}\;}_{30}^{k}\;=\;\frac{\hat{{\sum \#\;HBP\;cases}_{30}^{ik}}}{{POP\;Gender}_{30}}\;(Status\;quo)$$ (17)

We reduced the weighted gender-hypertension prevalence rate (Eq 16) by 17% to project new prevalence rate by gender under scenario II.

$$\overset{̿}{{{Scenario\;II\;HBP\;Prev}_{30}}^{k}}\;=\;[\hat{{{WPrev}_{HBP\;}}_{30}^{k}}*(1-17\%)]$$ (18)

We multiplied the new HBP prevalence rate with 2030 gender population and estimated new HBP cases by gender in 2030 (Scenario II).

$$\overset{̿}{{{Scenario\;II\;HBP\;case\;}_{30}}^{k}}\;=\;[\overset{̿}{{{Scenario\;II\;HBP\;Prev}_{30}}^{k}}*{POP}_{30}^{k}]$$ (19)

Finally, we distributed new gender-HBP cases to corresponding age groups, based on age-groups-shares in total gender-hypertension cases in 2030 status quo.

$$\overset{̿}{{Scenario\;II\;HBP\;cases\;}_{30}^{ik}}\;=\;[\overset{̿}{{Scenario\;II\;HBP\;case\;}_{30}^{k}}*(\frac{\hat{{HBP\;cases\;}_{30}^{ik}}}{\hat{{HBP\;cases\;}_{30}^{k}}}]$$ (20)

We applied a similar method for the projection of the number of hypertensive people in treatment and control in 2030 Scenario II. According to the MoH survey, 1.2% adult population in 2011 claimed that they were aware of hypertension but were not in treatment [19]. We assumed that this percentage will likely to be the case in 2030. In consequence, we estimated the number of people aware of their Hypertension by adding those already in treatment and the 1.2% of the adult population (15+ years).

In scenario II, when the hypertension prevalence rate reduced, we projected that there will be 2.8 million fewer people with hypertension (i.e. hypertension is under control with SBP<140 mmHg or DBP<90 mmHg). However, we assumed that these individuals will likely to carry the risk of being hypertensive and would stay under the hypertension treatment, in addition to the hypertensive people. Therefore, the projected number of people under treatment will be higher in the scenario II than that of the projected people in the status quo. Furthermore, the people under treatment will be aware of the disease. Therefore, we projected the number of hypertension awareness in 2030 by adding hypertensive people in treatment and the 1.2% of adults who are aware of hypertension but not in the treatment.

#### Economic benefits of reducing hypertension associated deaths and disabilities

In the cost and benefit analysis, it is a common practice to assign DALYs a monetary value. There is a great deal of literature discussing how to value DALY as a monetary base [27, 32, 35, 36, 37, 38, 39, 40]. The WHO Report of the Commission on Macroeconomics and Health [37] argues that the standard methodology for the DALY follows the human capital approach and that the value of an extra year of healthy life, resulting e.g. from successfully treating a disease, is worth more than the extra market income that will be earned in the year. Hence, averted DALY may be monetized using GDP per capita. It is also a common practice to conduct sensitivity analysis by assessing DALY with different fractions of GDP (1/3^rd^ of GDP to 3 or 5 GDP per DALY). For example, a recent study by Nugent et al. [27] values a DALY equal to 3 GDP/capita in Bangladesh. We value a DALY with a real GDP/capita (inflation-adjusted based on 2015 = 100) in national currency. We estimate real GDP/capita by dividing current GDP/capita with the GDP deflator between 2015 and 2023. Then we apply the same technique as the CPI projection to project GDP per capita and the GDP deflator between 2024 and 2030. Here we assume constant growth in GDP/capita and GDP deflator between 2024 and 2030. Since the IMF’s projections show almost a steady increase in GDP/capita and the GDP deflator during the last 5 years (2019–2023), we assume that a constant growth can continue until 2030. This method of projection is also applied for the real gross annual income per gender. The projected monetary values are, then applied on DALYs averted to project the economic benefits of the reduced burden of hypertension.

#### Projecting the cost of hypertension treatment in 2030

In 2016, we collected prices of frequently prescribed antihypertensive medications and estimated the annual average cost for the medications. We adjusted the price for the period 2015 to 2023 using the consumer price index (CPI) obtained from the IMF Economic Outlook and holding 2015 as the base year. We applied the average change in CPI between 2019 and 2023 to derive CPIs between 2024 and 2030, and estimated the real price of the average price of antihypertensive medicine using 2015 as the base year (i.e. CPI 2015 = 100).

The NIH Report [5] discussed the impact of pharmacologic treatment for hypertensive cases and indicated that since most hypertensive patients will require two or more antihypertensive medications to achieve their BP goals, addition of a second drug from a different class should be initiated when use of a single agent in adequate doses fails to achieve the goal [5]. PatenT2 survey revealed the percentage of hypertensive people who used a few antihypertensive medications (1 to 4 medicines a day) in 2012; i.e., among antihypertensive medication users, 38% uses one, 44% uses two, 16% uses three and 2.3% uses four medication a day. In our projections, we maintained those percentages constant. Furthermore, NIH 7^th^ report indicates that “once the antihypertensive drug therapy is initiated, most patients should return for follow up and adjustment of medications at monthly intervals or until the BP goal is reached. More frequent visits will be necessary for patients with stage 2 hypertension or with complicating comorbid conditions. After BP is at goal and stable, follow up visits can usually be at 3-to-6-month intervals" ([5], page 32). Since we have no information about the number of physician consultations, we assumed that hypertensive people will visit physicians as much the number of medications they use a year. We assumed that as the hypertension prevalence reduces in 2030, those ex-hypertensive people will stay under hypertension treatment and will continue their medication to keep their blood pressure under control.

### Results

We estimated that 25% of adult population (15+ years) or 14.5 million adults were hypertensive in 2015 (Table 4). In status quo, i.e., if the hypertension prevalence remaining constant until 2030, it is projected that 17.4 million will have hypertension, due to increases in population between 2015 and 2030. Under the status quo, we projected that about 55% of hypertensive people will be under the treatment but that would increase to 74% under Scenario II. Awareness will increase from 10.4 million in status quo to 11.6 million in scenario II.

**Table 4:** Hypertensive population, prevalence, awareness, treatment, and control (000).

		**2015**		**2030** **Status quo**		**2030** Scenario II*	
Population (15+)	Male	28,278		34,133			
	Female	29,69		35,985			
	Total	58,247		70,118			
People with Hypertension * & The Prevalence rate (%)	Male	6,267	22%	7,565	22%	6,341	18.6%
	Female	8,216	27%	9,866	27%	8,270	23.0%
	**Total**	**14,484**	**25%**	**17,431**	**25%**	**14,611**	**20.8%**
Hypertension in Control (number) & % share in under Treatment	Male	1,518	54%	1,832	54%	2,183	54%
	Female	2,600	51%	3,122	51%	3,434	51%
	**Total**	**4,117**	**52%**	**4,953**	**52%**	**5,617**	**52%**
People under Treatment & % share in hypertensive people	Male	2,780	45%	3,379	45%	4,029	64%
	Female	5,133	62%	6,164	62%	6,712	81%
	**Total**	**7,933**	**55%**	**9,542**	**55%**	**10,741**	**74%**
Awareness (number)** & % of population	Male	3,102	11%	3,744	11%	4,394	11%
	Female	5,529	18%	6,640	18%	7,188	20%
	**Total**	**8,632**	**15%**	**10,384**	**15%**	**11,582**	**16.5%**

Source: Authors’ calculation.

* number of HBP cases include those 1.2% population who were aware of HBP but not in treatment. Those 1.2% awareness were not included in the calculation when the 17% reduction in HBP prevalence was estimated.

** number of awareness estimation include those under treatment and those 1.2% population who were aware of HBP but not in treatment.

#### The number of hypertension attributable deaths and DALYs in 2015 and 2030

Hypertension was responsible for 54,252 deaths or about 14.4% of 376 thousand total adult deaths in 2015 [2, 41]. The burden was higher in women 28,612 deaths as compared with that of men with 25,640 deaths (Table 5). About 22% or 11,682 deaths occurred among economically active age population age 30–64 years old.

**Table 5:** Estimated deaths associated with hypertension in 2015.

Year (2015)	Male	Female	Total
**Total (30+ age population)**			
Total All-causes of Deaths (000)	204	172	376.1
Total Hypertension Deaths (000)	25.6	28.6	54.3
Hypertension attributable deaths as % all causes deaths in 30+ population	12.6	16.6	14.4
**Weighted Death Rate/100,000 population**			
30+ years-population	173.6	140.0	
30–64 years-population	67.4	19.25	
**30–64 Population**			
Total All-cause of Deaths (000)	63.3	30.2	7.1
Number of hypertension attributable Deaths (000)	7.75	3.9	11.7
% share in deaths in 30–64 population	31	17.5	24.9

Table 6 presents the projected total deaths and hypertension attributable deaths in 2030 in the status quo and Scenario II. In the status quo conditions, we projected 490.4 thousand adult deaths by all causes in 2030 and 91 thousand adults (30+ years) will die from hypertension. The hypertension attributable deaths will be higher in women with 49.8 thousand deaths compared to that of men with 41.3 thousand deaths. In scenario II, we estimated 466.1 thousand adult deaths by all causes; 24.3 thousand fewer adult deaths compared to the status quo scenario. When we attributed this to hypertension, and deduct from the hypertension deaths in status quo, the estimated total hypertension attributable deaths is 66.9 thousand in scenario II. Accordingly, we estimated that 13.6 thousand and 10.7 thousand hypertension-deaths in male and in female will be averted in 2030, respectively. Under the status quo and scenario II projections, we estimated that hypertension will claim 16.5 thousand and 12.6 thousand lives in economically active population, respectively. This entails that 16.2% or 3.9 thousand out of 24.3 thousand deaths averted will be among economically active 30–64 years of age population (Table 6). These results, on the one hand, suggest that hypertension is still an important killer among the economically active population. The deaths by this subgroup, on the other hand, will reduce by 24% from 16.5 thousand (status quo) to 12.6 thousand in the scenario II.

**Table 6:** Projected total and hypertension deaths in 2030.

	Status Quo			Scenario II		
	Male	Female	Total	Male	Female	Total
**30+ years population**						
Total All-causes Deaths (000)	267.3	223.1	490.4	253.7	212.4	466.1
Deaths averted (000) or (lives saved)				13.6	10.7	24.3
Number of Hypertension Deaths (000) *	41.3	49.8	91.2	27.7	39.2	66.9
**30–64 years-population**						
Total All-causes of Deaths (000)	83.0	39.1	122.1	80.2	38.0	118.2
Deaths averted (000) or (lives saved)				2.8	1.1	3.9
Number of Hypertension Deaths (000)	11.1	5.4	16.5	8.3	4.3	12.6

*New hypertension deaths in Scenario II is estimated by subtracting hypertension deaths in status quo from the deaths averted from total population.

In 2015, the burden of hypertension deaths was 987 thousand DALYs which was equivalent to 29.3 billion TL and 37.8 billion TL as one DALY was valued at GDP/capita and gross income/capita, respectively (Table 7). About half of the burden (451 thousand out of 987 thousand DALYs) will be borne by the economically active population (30–64 years old population) (Table 7).

**Table 7:** Burden of DALYs in 2015.

	DALYs (000)	DALYs (Billion) TL	
		Using GDP per capita	Using gross income per capita
30+ age population	987.0	29.3	37.8
30–64 age group	451.0	13.4	17.2

#### Projected total treatment cost

According to the Turkish MoH statistics, the total ambulatory care expenditure in 2015 was 8.9 billion TL [42], equivalent to 152.1 TL (USD 56.3) per ≥15 year adult population. We predicted that ambulatory care expenses per 15+ year adult population will increase to 582.9 TL in real terms, adjusting for inflation (CPI, 2015 = 100). The inflation adjusted average antihypertension medication (30 pills) price was estimated at 183.3 TL ($67) per year in 2015, which increased to 665.5 TL per year in real terms by 2030. The estimates entail that the cost for hypertension treatment was 4.9 billion TL, (2.7 billion TL for medication and 2.2 billion TL ambulatory care (physician consultation) constituting about 5.5% of total health care expenditures in 2015. We projected that the cost will increase by almost five-fold in 2030 to 21.7 billion TL in real terms (11.59 billion TL for medication and 10.15 billion TL for ambulatory care) under the status quo. In Scenario II, the projected treatment cost increased by 2.7 billion TL compared to the status quo scenario, i.e. from 21.7 billion TL to 24.46 billion TL between status quo and scenario II (Table 8).

**Table 8:** Estimated hypertension treatment costs, 2015 and 2030.

	2015	2030		
		**Status quo**	Scenario II*	
Average medication (TL)	183.3^§^	665.5^¶^	665.5	
Ambulatory care/adult (TL)	152.1	582.9	582.9	
	**Cost in Billion TL**			Additional cost in Billion TL (Scenario II—Status Quo)
Annual medication	2.7	11.6	13.0	1.4
Annual ambulatory care	2.2	10.2	11.4	1.3
Total treatment	4.9	21.7	24.5	2.7

Note:

^§^Data provides the percentage of hypertensive people using a number of medications (1–4). In our analysis, we assume that each medication costs on average 17 TL per month in 2016.

^¶^ We projected the conversion rate between USD and TL based on data on the IMF WEO database, where the conversion rate is projected to increase by 5.5% per annum over the period 2018–2022. In our projection for 2030, we kept the rate constant at 5.5%.

* a 17% reduction in hypertension prevalence in 2030.

#### Comparison of economic benefits and costs

Table 9 presents the estimated the economic burden of hypertension in DALYs in the status quo and scenario II. We projected that 392 thousand DALYs will be averted in Scenario II, i.e. 1,561 thousand DALYs in status quo compared to 1169 thousand in Scenario II. The economic burden of hypertension attributable DALYs will reduce between 18 billion TL and 23.2 billion TL when a DALY is valued at GDP per capita and gross income per capita, respectively.

**Table 9:** Predicted costs and benefits of hypertension management in 2030.

	DALYs (000)	DALYs in (Billion) TL valued by		Savings in DALYs averted (Billion TL)		Additional medical costs (Billion TL)	Benefit/cost
Column #	(1)	(2)		(3)		(4)	(5) = (3)/(4)
		Real GDP/ capita	Real Gross Income/ Capita	Real GDP/ capita	Real Gross Income/ capita		
**Status quo**	1,561	71.8	92.6	-----	-----		
**Scenario II**	1,169	53.7	69.4	18.0	23.2	2.7	**6.7–8.6**

In the Scenario II, the projected cost in 2030 associated with hypertension treatment increased by an additional 2.7 billion TL from 21.7 billion TL in status quo to 24.5 billion TL in Scenario II. Accordingly, the economic benefit (savings) of the reduced burden of hypertension associated DALYs (i.e. DALYs averted in TL) will exceed the additional hypertension treatment cost by 6.7 and 8.6 folds, respectively.

## Conclusion

Hypertension is a significant public health issue in Turkey. Diseases associated and attributed to hypertension already put a heavy burden on MoH’s budget. A study by Pektas et al. [43] revealed that Turkish Insurance Agency reimbursed 1.3 billion TL (USD 663 million) for antihypertensive medication alone in 2013. Consequently, the MoH initiated a national salt reduction plan in 2011 and the second plan (2016–2021) was initiated in 2016 that aimed to reduce the salt consumption by 30%. The projections suggest that hypertension prevention and management is a good investment with a high economic benefit that outweighs the costs associated with extended hypertension treatment. To this effect, a reduction in population-wide salt consumption is no small difference concerning averting deaths and saving lives in adult and economically active populations.

It is important to emphasize a few issues about the models and the results. The projections are based on some assumptions; and therefore, the results may need to be evaluated as indicative. For example, predictions for the status quo are made by keeping some of the 2015 data constant. Specifically, the age-adjusted weighted death rates for the hypertensive population are held constant between 2015 and 2030 when we project the deaths associated with hypertension. It is difficult to project how the hypertension trend will take a course for the next 15 years. For example, the death rates and the number of deaths from hypertension by IHME decrease between 1990 and 2005 for the age groups by genders, and then show an increasing trend between 2010 and 2015. Furthermore, we assume that projected deaths averted between the status quo and the Scenario II are attributable to hypertension only. However, it is very likely that some of the averted deaths can be attributed to elevated blood pressure and other risk factors. As a result, the projected hypertension deaths in 2030 (Scenario II) may be overestimated. On the other hand, we believe that the cost projections for both the antihypertensive medication and the physician consultations are likely to be overestimated. This due to our assumption that, notwithstanding a reduction of the hypertension prevalence by 17%, the individuals who are not part of the hypertensive population in 2030 will continue to stay under the hypertension treatment. It is very likely that some of these individuals may opt out from the treatment (antihypertensive medication and physician consultation) and seek for healthy life changes to keep controlling their hypertension. In consequence, the cost benefits ratio can be considered conservative.

Despite these limitations, these results provide some degree of direction for the benefits of reducing death and prevalence rates despite increases in hypertension treatment; and the salt reduction program is one of the ways to achieve that. As an increasing number of countries focuses on salt reduction programs [8], some countries such as the United Kingdom [44, 45], Finland [46, 47, 48] and Japan [49] successfully reduced population-wide salt intake and achieved reduction in blood pressure and cardiovascular mortality [50]. Consequently, health services benefited from significant savings [28]. This study suggests that population-wide hypertension prevention and management is a win-win situation for public health and Turkish health care system as the benefits of hypertension management (reduced prevalence and deaths) outweigh the costs associated with extending hypertension treatment.

## References

[1] World Health Organization. A global brief on hypertension: silent killer, global public health crisis: World Health Day 2013. World Health Organization; 2013.

[2] IHME 2018. Global Health Data Exchange. Global Burden of Disease Study 2016 (GBD 2016) Data Resources. Institute for Health Metrics and Evaluation, University of Washington. http://ghdx.healthdata.org/gbd-2016

[3] Mathers CD, Loncar D. Updated projections of global mortality and burden of disease, 2002–2030: data sources, methods and results. Geneva: World Health Organization 2005 10.

[4] Marks JS., McQueen DV. Chronic Disease In: Critical Issues in Global Health, ed. Koop CE., Pearson CE., Schwarz MR. 2002 Pp. 117–26. San Francisco: Jossey-Bass.

[5] National Institute of Health (NIH). The Seventh Report of the Joint National Committee (JNC7) on Prevention, Detection, Evaluation, and Treatment of High Blood Pressure; US Department of Health and Human Services, NIH, National Heart, Lung, and Blood Institute, National High Blood Pressure Education Program. NIH publication; August 2004. https://www.ncbi.nlm.nih.gov/books/NBK9630/pdf/Bookshelf_NBK9630.pdf 20821851

[6] Erdem Y, Akpolat T, Derici Ü, Şengül Ş, Ertürk Ş, Ulusoy Ş, Altun B, Arıcı M. Dietary sources of high sodium intake in Turkey: SALTURK II. Nutrients. 2017;9(9):933. 10.3390/nu9090933 PMC5622693 28837102

[7] Erdem Y, Arici M, Altun B, Turgan C, Sindel S, Erbay B, Derici U, Karatan O, Hasanoglu E, Caglar S. The relationship between hypertension and salt intake in Turkish population: SALTURK study. Blood pressure. 2010 10 1;19(5):313–8. 10.3109/08037051003802541 20698734

[8] Trieu K, Neal B, Hawkes C, Dunford E, Campbell N, Rodriguez-Fernandez R, Legetic B, McLaren L, Barberio A, Webster J. Salt reduction initiatives around the world–a systematic review of progress towards the global target. PloS one. 2015 7 22;10(7):e0130247 10.1371/journal.pone.0130247 26201031 PMC4511674

[9] Cutler JA, Follmann D, Allender PS. Randomized trials of sodium reduction: an overview. The American journal of clinical nutrition. 1997 2 1;65(2):643S–51S. 9022560 10.1093/ajcn/65.2.643S

[10] Stamler R. Implications of the INTERSALT study. Hypertension. 1991 1;17 (1_supplement):I16. 1986996 10.1161/01.hyp.17.1_suppl.i16

[11] Cook NR, Cohen J, Hebert PR, Taylor JO, Hennekens CH. Implications of small reductions in diastolic blood pressure for primary prevention. Archives of Internal Medicine. 1995 4 10;155(7):701–9. 7695458

[12] Whelton PK, He J, Appel LJ, Cutler JA, Havas S, Kotchen TA, et al Primary prevention of hypertension: clinical and public health advisory from The National High Blood Pressure Education Program. Jama. 2002 10 16;288(15):1882–8. 10.1001/jama.288.15.1882 12377087

[13] Sacks FM, Svetkey LP, Vollmer WM, Appel LJ, Bray GA, Harsha D, et al Effects on blood pressure of reduced dietary sodium and the Dietary Approaches to Stop Hypertension (DASH) diet. New England journal of medicine. 2001 1 4;344(1):3–10. 10.1056/NEJM200101043440101 11136953

[14] World Bank. Health Nutrition and Population Database: Population estimates and projections. 2018. Avaialable from: http://databank.worldbank.org/data/reports.aspx?source=Health-Nutrition-and-Population-Statistics:-Population-estimates-and-projections

[15] SALTURK II. 2012. http://turkhipertansiyon.org/tuz_280512.php & www.turkhipertansiyon.org/pdf/salt_160608.ppt

[16] Global Adult Tobacco Survey (GATS) comparison fact sheet: Turkey 2008 and 2012. http://www.who.int/tobacco/surveillance/survey/gats/gats_turkey_2008v2012_comparison_fact_sheet.pdf

[17] Republic of Turkey Ministry of Health. Global Adult Tobacco Survey Turkey 2012. Ministry of Health Publication No: 948. 2014. Ankara.

[18] Dastan I, Erem A, Cetinkaya V. Awareness, treatment, control of hypertension, and associated factors: Results from a Turkish national study. Clinical and Experimental Hypertension. 2018 1 2;40(1):90–8. 10.1080/10641963.2017.1334797 28686064

[19] Republic of Turkey Ministry of Health. Chronic Diseases and Risk Factors Survey in Turkey. 2013. Ministry of Health Publication No: 909. Republic of Turkey Ministry of Health Turkish Public Health Institution, Chronic Diseases, Elderly Health, and Disabled Department.

[20] Satman I, Yilmaz T, Sengül A, Salman S, Salman F, Uygur S, Bastar I, Tütüncü Y, Sargin M, Dinççag N, Karsidag K. Population-based study of diabetes and risk characteristics in Turkey: results of the turkish diabetes epidemiology study (TURDEP). Diabetes care. 2002 9 1;25(9):1551–6. 10.2337/diacare.25.9.1551 12196426

[21] Satman I, Omer B, Tutuncu Y, Kalaca S, Gedik S, Dinccag N, Karsidag K, Genc S, Telci A, Canbaz B, Turker F. Twelve-year trends in the prevalence and risk factors of diabetes and prediabetes in Turkish adults. European journal of epidemiology. 2013 2 1;28(2):169–80. 10.1007/s10654-013-9771-5 23407904 PMC3604592

[22] Altun B, Arici M, Nergizoglu G, Derici Ü, Karatan O, Turgan Ç, Sindel S, Erbay B, Hasanoglu E, Çaglar S. Prevalence, awareness, treatment and control of hypertension in Turkey (the PatenT study) in 2003. Journal of hypertension. 2005 10 1;23(10):1817–23. 16148604 10.1097/01.hjh.0000176789.89505.59

[23] PatenT I 2003: Turkish Society of Hypertension and Renal Diseases. http://turkhipertansiyon.org/prevelans_calismasi.php

[24] Paten T II. 2012. Turkish Society of Hypertension and Renal Diseases. http://turkhipertansiyon.org/prevelans_calismasi_2.php

[25] Arici M, Turgan C, Altun B, Sindel S, Erbay B, Derici U, Karatan O, Erdem Y, Hasanoglu E, Caglar S. Hypertension incidence in Turkey (HinT): a population-based study. Journal of hypertension. 2010 2 1;28(2):240–4. 10.1097/HJH.0b013e328332c36b 19809361

[26] TUIK 2015. Saglik istatistikleri yilligi: Annual health statistics. http://ekutuphane.sagem.gov.tr/kitaplar/saglik_istatistikleri_yilligi_2015.pdf

[27] Nugent R, Brower E, Cravioto A, Koehlmoos T. A cost-benefit analysis of a National Hypertension Treatment Program in Bangladesh. Preventive medicine. 2017 12 1;105:S56–61. 10.1016/j.ypmed.2017.08.014 28827074

[28] Murray CJ, Lauer JA, Hutubessy RC, Niessen L, Tomijima N, Rodgers A, Lawes CM, Evans DB. Effectiveness and costs of interventions to lower systolic blood pressure and cholesterol: a global and regional analysis on reduction of cardiovascular-disease risk. The Lancet. 2003 3 1;361(9359):717–25. 10.1016/S0140-6736(03)12655-4 12620735

[29] Asaria P, Chisholm D, Mathers C, Ezzati M, Beaglehole R. Chronic disease prevention: health effects and financial costs of strategies to reduce salt intake and control tobacco use. The Lancet. 2007 12 15;370(9604):2044–53. 10.1016/S0140-6736(07)61698-5 18063027

[30] Murray CJL, Lopez AD, eds.. The global burden of disease: a comprehensive assessment of mortality and disability from diseases, injuries and risk factors in 1990 and projected to 2020 Global Burden of Disease and Injury Series, Vol. 1 Cambridge: Harvard University Press; 1996.

[31] Murray CJL., Lopez A. Global Health Statistics Cambridge, Harvard University Press (Global Burden of Disease and Injury Series, Vol. 2). 1996.

[32] Murray CJ, Lopez AD. Global mortality, disability, and the contribution of risk factors: Global Burden of Disease Study. The lancet. 1997 5 17;349(9063):1436–42. 10.1016/S0140-6736(96)07495-8 9164317

[33] Norman R, Bradshaw D, Schneider M, Pieterse D, Groenewald P. Revised burden of disease estimates for the comparative risk factor assessment, South Africa 2000. Cape Town: Medical Research Council 2006.

[34] World Health Organization. The global burden of disease: 2004 update. Geneva 2008 https://www.who.int/healthinfo/global_burden_disease/GBD_report_2004update_full.pdf?ua=1

[35] World Bank, World Development Report 1993; investing in health. Oxford Oxford University Press, 1993.

[36] Jamison DT, Breman JG, Measham AR, Alleyne G, Claeson M, Evans DB, Jha P, Mills A, Musgrove P, editors. Priorities in health. The World Bank; 2006 4 2. 21089239

[37] World Health Organization (WHO). Macroeconomics and Health: Investing in Health for Economic Development Report of the Commission on Macroeconomics and Health. Geneva, Switzerland 2001.

[38] Robinson LA, Hammitt JK, Chang AY, Resch S. Understanding and improving the one and three times GDP per capita cost-effectiveness thresholds. Health Policy and Planning. 2016 7 24;32(1):141–5. 10.1093/heapol/czw096 27452949

[39] Augustovski F, Colantonio LD, Galante J, Bardach A, Caporale JE, Zárate V, et al Measuring the benefits of healthcare: DALYs and QALYs–Does the choice of measure matter? A case study of two preventive interventions. International journal of health policy and management. 2018 2;7(2):120. 10.15171/ijhpm.2017.47 PMC5819372 29524936

[40] Edwards C. Social Cost-Benefit Analysis–the available evidence on drinking water. Valuing Water–Valuing Well-being: A Guide to Understanding the Costs and Benefits of Water Interventions. 2008.

[41] TUIK Causes of Death Statistics 2018. http://www.tuik.gov.tr/PreHaberBultenleri.do?id=24572

[42] TUIK Health Statistics 2016. https://biruni.tuik.gov.tr/demografiapp/olum.zul

[43] Pektas MB, Aldemir M, Pektas A, Akci O, Dogan I. Would Anti-Hypertensive Treatment be a Risk Factor for National Health Budget of Turkey in 2023?. Medicine Science.;4(4):2813–24.

[44] Sadler K, Nicholson S, Steer T, Gill V, Bates B, Tipping S, Cox L, Lennox A, Prentice A. Assessment of dietary sodium levels among adults (aged 19–64) in England, 2011. London: Department of Health 2012.

[45] He FJ, Pombo-Rodrigues S, MacGregor GA. Salt reduction in England from 2003 to 2011: its relationship to blood pressure, stroke and ischaemic heart disease mortality. BMJ open. 2014 4 1;4(4):e004549 10.1136/bmjopen-2013-004549 24732242 PMC3987732

[46] Karppanen H, Mervaala E. Sodium intake and hypertension. Progress in cardiovascular diseases. 2006 9 1;49(2):59–75. 10.1016/j.pcad.2006.07.001 17046432

[47] Laatikainen T, Pietinen P, Valsta L, Sundvall J, Reinivuo H, Tuomilehto J. Sodium in the Finnish diet: 20-year trends in urinary sodium excretion among the adult population. European journal of clinical nutrition. 2006 8;60(8):965 10.1038/sj.ejcn.1602406 16482074

[48] Pietinen P, Valsta LM, Hirvonen T, Sinkko H. Labelling the salt content in foods: a useful tool in reducing sodium intake in Finland. Public health nutrition. 2008 4;11(4):335–40. 10.1017/S1368980007000249 17605838

[49] Sasaki N. The salt factor in apoplexy and hypertension: epidemiology studies in Japan. Prophylactic approach to hypertensive diseases. 1979:467–74.

[50] He FJ, MacGregor GA. Salt and sugar: their effects on blood pressure. Pflügers Archiv-European Journal of Physiology. 2015 3 1;467(3):577–86. 10.1007/s00424-014-1677-x 25547872

